# Traits underlying community consequences of plant intra-specific diversity

**DOI:** 10.1371/journal.pone.0183493

**Published:** 2017-09-08

**Authors:** Luis Abdala-Roberts, Riley Pratt, Jessica D. Pratt, Kailen A. Mooney

**Affiliations:** 1 Departamento de Ecología Tropical, Campus de Ciencias Biológicas y Agropecuarias, Universidad Autónoma de Yucatán, Mérida, Yucatán, México; 2 University of California, Irvine, Department of Ecology and Evolutionary Biology, Irvine, California, United States of America; 3 Irvine Ranch Conservancy, Irvine, California, United States of America; Rutgers The State University of New Jersey, UNITED STATES

## Abstract

A plant’s performance and interactions with other trophic levels are recorgnized to be contingent upon plant diversity and underlying associational dynamics, but far less is known about the plant traits driving such phenomena. We manipulated diversity in plant traits using pairs of plant and a substitutive design to elucidate the mechanisms underlying diversity effects operating at a fine spatial scale. Specifically, we measured the effects of diversity in sex (sexual monocultures vs. male and female genotypes together) and growth rate (growth rate monocultures vs. fast- and slow-growing genotypes together) on growth of the shrub *Baccharis salicifolia* and on above- and belowground consumers associated with this plant. We compared effects on associate abundance (# associates per plant) vs. density (# associates per kg plant biomass) to elucidate the mechanisms underlying diversity effects; effects on abundance but not density suggest diversity effects are mediated by resource abundance (i.e. plant biomass) alone, whereas effects on density suggest diversity effects are mediated by plant-based heterogeneity or quality. Sexual diversity increased root growth but reduced the density (but not abundance) of the dietary generalist aphid *Aphis gossypii* and its associated aphid-tending ants, suggesting sex mixtures were of lower quality to this herbivore (e.g. via reduced plant quality), and that this effect indirectly influenced ants. Sexual diversity had no effect on the abundance or density of parasitoids attacking *A*. *gossypii*, the dietary specialist aphid *Uroleucon macolai*, or mycorrhizae. In contrast, growth rate diversity did not influence plant growth or any associates except for the dietary specialist aphid *U*. *macolai*, which increased in both abundance and density at high diversity, suggesting growth rate mixtures were of higher quality to this herbivore. These results highlight that plant associational and diversity effects on consumers are contingent upon the source of plant trait variation, and that the nature of such dynamics may vary both within and among trophic levels.

## Introduction

A plant’s performance and interactions with other trophic levels are recognized to be influence its neighbours. The study of such dynamics have adopteded the differing, but complementary perspectives of associational effects and diversity effects. Herbivore abundance and damage may be increased or decrease by plant neighbors, termed associational susceptibility and resistance, respectively, with these outcomes depending on the identities and traits of the plants and their associated herbivores [1,2]. Plant associational effects can also influence higher trophic levels. For example, a plant’s herbivores or predators may be influened by spillover of herbivores or predators from neighbouring plants, but may also arise from the effects of neighbouring plants on host plant plant growth or quality [3–5]. Such associational effects on higher trophic levels can in turn influence a plant’s growth and reproduction [6]. The aggregated of such associational effects among a set of plant neighbors are in turn the basis for the higher-order effects of plant diversity. Here, polyclutures of co-occurring genotypes or species (intra- and inter-specific diversity, respectively) are compared to monocultures to test for non-additive effects with respect to plant performane and interactions with higher trophic levels (reviewed in [7]). Accordingly, plant heterogeneity can affect community and ecosystem processes across multiple trophic levels with a common set of mechanisms operating across different scales [8].

Plant intra-specific or genotypic diversity has been shown to boost primary productivity [9], increase arthropod diversity [9–11], and alters plant-herbivore and herbivore-predator interactions [12–14]. Despite the documented importance of plant intra-specific diversity and its underlying associational effects, the mechanisms underlying such effects are poorly understood. In past studies, genotypes have been sampled at random and without regard to particular traits (but see [15]) and, as a result, such studies fail to address the plant traits mediating observed patterns [16,17]. Although *a posteriori* correlations can be made between plant traits and effects on higher trophic levels, this approach may be burdened by low statistical power if many traits are studied and may suffer from increased risk of spurious associations. In addition, intra-specific effects are in many cases likely mediated by simultaneous effects of multiple plant traits, yet relatively little is known about the relative importance and independent effects of such traits. Accordingly, the experimental manipulation of specific traits of a focal species is of fundamental importance towards developing a mechanistic understanding of diversity effects [15,18].

Plant genetic diversity may influence associated communities through several complementary mechanisms. First, genotype resource partitioning and facilitation may lead to increased plant biomass (e.g. [9, 10, 13]), thus increasing plant-based resources–i.e. resource abundance–with concomitant increases in associate abundance [9, 19]. Second, increased plant-based heterogeneity may influence associate behavior (e.g. host plant selection or residence times; [2, 14]). And third, diversity may alter plant traits associated with plant quality (e.g. plant defenses in the case of herbivores; [20]). Diversity effects acting through changes in plant heterogeneity or quality should influence associates independently of changes in plant-based resource abundance, and such effects should thus be detectable as changes in associate density, i.e. effects on associates occurring having accounted for any change in plant biomass [14, 21].

Plant genetic variation in growth rate represents an axis of phenotypic variation underlain by multiple correlated traits that may be an important driver of plant intra-specific diversity effects on plant productivity and consumers. On the one hand, genetically-determined differences in growth rate may promote increased plant productivity because contrasting growth rates are associated with different nutrient acquisition strategies [22]. It is also possible that competition among genotypes with differing growth rate is weaker in mixtures than in monoculture, particularly for fast-growing genotypes, and this would lead to increased productivity in mixtures due to dominance effects by productive genotypes [15]. Greater plant biomass should in turn cascade-up to positively influence consumer abundance [9,10]. On the other hand, differences in growth rate among genotypes may also influence plant quality when, for example, higher growth in neighbourhoods with genotypic mixtures leads to lower investment in plant defenses if growth and defenses trade off [13,23]. Such effects on plant traits are expected to influence consumer behavior (e.g. feeding rates, foraging time) but are not explained by differences in plant abundance per se. Alternatively, differences in growth rate may influence consumer behavior through processes occurring independently of plant-plant interactions if fast- and slow-growing genotypes vary in traits influencing plant heterogeneity and thus consumer recruitment [24].

Plant genetic differences between sexes represent another important proxy of correlated trait variation, which might act as a source of intra-specific diversity effects [18]. Dioecy is present in 6–10% of all angiosperm species [25–27], is most often genetically determined [28], and plant sex is associated with a substantial degree of ecological dimorphism [29]. Females frequently invest more in reproduction than males or hermaphrodites [30,31], grow more slowly, and invest more in traits conferring herbivore resistance [26,32]. These phenotypic differences may influence above- [29,33,34], and below-ground [35,36] plant-associated communities. Accordingly, as for plant growth, phenotypic differences between sexes may positively influence consumer abundance through increases in plant growth if plants of sexes exhibit complementary resource acquisition strategies. Alternatively, variation in sex may influence consumer behavior due to changes in plant quality underlain by growth-defense trade-offs or via associational effects influencing consumer recruitment independently of plant-plant interactions. To our knowledge, only one study has tested for associational effects of plant sex and found no effect on levels of rust (*Melampsora* spp.) infection in the shrub *Salix viminalis* [18].

In a previous study with the dioecious shrub *Baccharis salicifolia* (Ruiz and Pav.) Pers. (Asteraceae), we reported on patterns of variation in above- and belowground plant associates among genetic lines of fast- and slow-growing male and female plants, namely two aphid species, aphid-tending ants, aphid parasitoids, and mycorrhizae [21]. Here we build from these findings using data from the same experiment to test for intra-specific diversity effects arising from differences in plant growth rate and sex. We documented variation in associate density (number of consumers per unit of plant biomass) to test for effects occurring through plant-based heterogeneity or quality (controlling for effects of plant biomass), and variation in associate abundance to test for effects occurring through both plant quality and abundance (including effects of plant biomass). Specifically, we address the following questions: (i) Does diversity in plant sex and growth rate influence plant productivity? (ii) What are the effects of sex and growth rate diversity on plant associates? (iii) Does sex and growth rate diversity influence associate abundance through changes in resource abundance (i.e. plant biomass) and/or associate density through changes in plant quality or heterogeneity? And finally, (iv) how do sex and growth rate diversity effects compare in their strength and mechanism of action on plant performance and associated communities? In so doing, this study provides unique mechanistic insight into the community-wide effects of intra-specific plant diversity.

## Materials and methods

### Natural history

*Baccharis salicifolia* is a woody perennial, dioecious shrub that is native to the Southwestern United States and Northern Mexico, and is usually found growing in riparian sites. Flowering takes place from March to May, which is when insect abundance is highest. In addition, this species frequently grows in high-density monospecific stands and multiple genotypes frequently co-occur at small spatial scales. At our study sites, found within and adjacent to the University of California San Joaquin Marsh Reserve (33°39’47” N, 117°51’7” W; CA, USA), the two most abundant aboveground herbivores of this species are the generalist aphid *Aphis gossypii* (Glover) and the specialist aphid *Uroleucon macolai* Blanchard [37]. *Aphis gossypii* (Glover) (*Aphis* hereafter) is a generalist herbivore that feeds on several important crops [38], whereas *U*. *macolai* (*Uroleucon* hereafter) is a dietary specialist that feeds only on *B*. *salicifolia* and one other *Baccharis* species [38]. In addition, at our study sites *Aphis* is frequently tended by the non-native argentine ant *Linepithema humile* Mayr, which feeds on the aphid’s honeydew [37,39]. In contrast, *Uroleucon* is not tended by ants. Parasitic wasps are the most common natural enemies of these aphid species (Hymenoptera: Braconidae) [37].

We previously documented the effects of plant sex (male vs. female genotypes) and growth rate (fast- vs. slow-growing genotypes) on plant associates based upon analysis of the same experiment and data used in the present study (S1 Table; [21]). Plant sex and growth rate did not affect *Uroleucon*. In contrast, male plants had higher abundances and densities of *Aphis* and its associated parasitoids (Braconidae) and aphid-tending ants (*L*. *humile*). Fast-growing genotypes had higher abundances but not densities of *Aphis* and ants. Male genotypes had lower abundances and densities of mycorrhizal colonization than females, while fast-growing genotypes had higher abundances but lower densities of mycorrhizal colonization than slow-growing genotypes. In the present study, we document the consequences diversity in sex and growth for plant associates, reporting on complementary analyses of the same experiment to compare mixtures (pairs) of plants that are either homogeneous (monocultures) or diverse (polycultures) with respect to plant growth rate and sex.

### General approach

Assessing the consequences of ecological heterogeneity has beeen made with resepct to both associational effects and diversity effects. Studies on associational effects focus on how the performance of the individual are affected by neighbors. Such studies typically compare two plant types (e.g. genotypes, species) in monoculture and polyculture in order to quantify the two separate (and potentially asymetrical) effects of each plant type on the other (i.e. treatments of AA, BB, AB assess the associational effects of A on B and B on A). In contrast, studies on diversity effects focs on how heterogneity scales up to dermine community and ecoystems level processes. Such studies typically compare several (≥ 2) plant types and measure the net outcome of all pairwise associational effects, where monocultures are used to make polycultures predictions to compare with polyculture observations (i.e. treatments of AA and BB used to predict AB, these predictions compared with observations of AB). Such studies can range from fine-scale effects emanating from pairs of neighbouring plants to larger-scale effects emanating from many plant types [40]. A common set of processes thus underlie associational and diversity effects [8]. Because our goal was to investigate the consequences of plant heterogeneity in sex and growth rate for multi-trophic communities, we adopted the conceptual and analytical approach of diversity effects.

### Genotype propagation and selection

The procedures for genotype propagation and selection are also described in Abdala-Roberts (2016). All plants used in this experiment were collected from a “source” common garden consisting of genotypes from a natural population of *B*. *salicifolia* in the San Joaquin Marsh Reserve, from which wild-grown plants were randomly selected over a 35 ha area (the most distant plants were approximately 900 m apart). This source common garden and the present experiment were adjacent to each other and to the Marsh Reserve. Therefore, the spatial scale of these experiments and our sampling of the wild-grown genotypes were roughly equivalent, which resulted in a realistic assessment of plant intra-specific effects on consumers [41].

In February 2008, we collected shoot cuttings from 20 male and 20 female wild-grown plants from the UC San Joaquin Marsh Reserve under the permission of the reserve manager. The cutings were transplanted them to 1L pots containing a soil mixture of equal parts silica sand, redwood compost, peat moss, and pumice and grown in a greenhouse. In May 2008, cuttings were randomly planted out into an open area with 1.0 m separation among plants. Sample size ranged from eight to 13 cuttings per genotype (mean 11.5 ± 0.2; mode = 12), with a total of 459 plants. In December 2008, all plants were assessed for size by measuring the cumulative length of all shoots longer than 10 cm in length. All plants were initiated at the same size and time, thus any subsequent measurements of plant size represented an estimate of plant growth rate.

Across all genotypes, there was significant (3.7-fold) variation in growth rate (total cumulative plant length after 10 months of growth) between the fastest and the slowest-growing genotypes, as well as a weak effect of plant sex with females growing 8% faster than males [21]. The three fastest and four slowest growing male and female genotypes (N = 14 genotypes total) were identified based on the above results and cloned for the present study. A greater number of slow- than fast-growing genotypes (four vs. three for each sex, respectively) were used because of lower propagation success of the former. In the source common garden, the average growth of the six fast male and female genotypes was two-fold greater than the average growth of the eight slow male and female genotypes, but there was no significant difference in growth between males and females [21].

In April 2009, three cuttings were collected from each of the approximately 12 copies of each plant of the selected genotypes in the source common garden (ca. 36 cuttings from each of 14 genotypes). Having replicate copies of each genotype come from unique source plants eliminates non-genetic (maternal) effects. These cuttings were treated as described above, grown in perlite until early June when they were transplanted into individual pots, and then maintained in a greenhouse until November 2009 when they were planted into the field experiment.

### Experimental design

The experimental design is also described in Abdala-Roberts (2016). In November 2009, we planted *B*. *salicifolia* individuals in 10 separate 2 × 2 m plots, each covered by 2.4 × 2.4 m cages made of PVC pipe frames encased with 70% transparent lumite fabric, and 1.4 m spacing between plots. For five plots, these cages were open on one side, although aphids, their natural enemies (parasitoids, coccinellids), and aphid-tending ants gained access and were common in all plots. Each plot was in turn divided into nine planting locations, each with a pair of plants (18 plants per plot) (S1 Fig). Planting locations were on a three-by-three grid, with 0.67 m spacing among locations and with edge planting locations being 20 cm from the cage wall. Plants within a pair were transplanted into a single excavated hole with plant root masses touching. Upon plant excavation at the end of the experiment (for biomass measurements, see ahead), roots had spread laterally only approximately 5 cm from the original planting locations, suggesting that while plants within pairs interacted, belowground interactions among pairs was unlikely. Plant canopies within pairs were consistently touching whereas plant canopies among pairs only touched occasionally, and in all cases canopy contact was greater within than among pairs.

With respect to plant sex, pairs of plants were either two males or two females (sex monocultures) or two plants of different sex (sex polycultures), with three repetitions of each combination per plot (S1 Fig). Similarly, with respect to growth rate plant pairs were either two fast- or two slow-growing genotypes (growth monocultures) or a fast- and slow-growing genotype (growth polycultures), also with three repetitions of each combination per plot (S1 Fig). These plant sex and growth rate combinations were crossed for a total of nine treatment combinations, with one repetition of each treatment combination in each plot. Across the entire experiment there were 10 replicate pairs per treatment combination, 30 replicate pairs for each sex monoculture, sex polyculture, growth monoculture, and growth polyculture, and overall a total of 90 plant pairs and 180 plants. With respect to individual genotypes, these were assigned such that each replicate of a treatment consisted of unique genotypic combinations. Sample sizes varied based upon plant material available, ranging from 10 to 20 plants per genotype (median of 13), with the exception of one slow-growing female genotype for which sample size was five. All plots were irrigated daily for the first six weeks and as needed throughout the summer and early fall of 2010 (May-November) and were weeded every other month from January through June of 2010 and 2011. All plants were inoculated with approximately 10 adults of the two dominant aphid species (*A*. *gossypii* and *U*. *macoli*) in January 2010 and again in January 2011. Our design was substitutive, and did not address the separate effects of plant density (intra-plant type effect) and frequency (i.e. inter-plant type effect) [24] as doing so would have required replicating the current experiment at multiple densities (e.g. [4]) which was not logistically feasible based upon available resources.

### Insect sampling and plant biomass measurements

We sampled insects twice during the experiment, once in March 2010 and again in March 2011. We recorded the abundance of *Uroleucon*, *Aphis*, ants, and *Aphis* parasitoids by carefully examining the stems and leaves of each plant. Parasitoid abundance was estimated from the number of visibly parasitized aphids per plant. There was no detectable parasitism on *Uroleucon*. Plants were harvested in May 2011 (18 months after planting), and plant material was divided into above- (i.e. shoot) and belowground (i.e. root) biomass, dried and weighed. We estimated insect abundance (counts) and density, where the latter was estimated as the number of insects per kilogram of shoot dry biomass. For statistical analyses, we used the sum of biomass and the mean of insect (abundance or density) values across plants within each pair.

### Mycorrhizal colonization

Fine roots (0.5–1.0 g) were soaked in 10% KOH for 4–5 days and then transferred to a 1% HCl solution for one minute. Roots were stained by placing them in a 0.01% acid fuschin in 14:1:1 lactic acid: glycerol:diH_2_O for one day. To de-stain, samples were transferred to 14:1:1 lactic acid: glycerol:diH_2_O solution for one day. Percent root colonization by arbuscular mycorrhizal fungi was calculated by examining 100 random intersect points of a slide with stained fine roots on it. An intersection point was defined as any time an observer encountered a root sample. Transect lines ran vertically through the slide; an observer then completed enough transect lines until 100 intersection points were recorded. At every intersection point, the presence or absence of mycorrhizal colonization was recorded and we calculated percent root colonization (i.e. proportion of intersection points colonized), considered a proxy of density of mycorrhizal colonization. Mycorrhizal abundance was estimated as the amount of colonized root mass by multiplying the proportion of colonized points by the root dry mass (g). Although this estimate of abundance is based upon both fine and coarse root biomass and therefore overestimates the mass of colonized roots as only fine roots are colonized, it nevertheless provides a relative estimate for comparison among levels of diversity.

### Statistical analyses

We used general linear mixed models to test for the effects of sex diversity and growth rate diversity on plant growth (total biomass, shoot biomass, and root biomass), mycorrhizae, *Aphis*, *Uroleucon*, parasitoids, and ants using plant pair (i.e. monoculture or polyculture) as the unit of replication. We tested for effects on both the abundance (number of insects or hyphae; hereafter "abundance" models) and density (number of insects or hyphae/kg of dry weight; hereafter “density” models) of insects and mycorrhizae. All insect abundance and density models were performed using values per plant averaged across years, as we found no evidence of inter-annual variation in sex or growth rate diversity effects for any group of insects (non-significant year × sex diversity and year × growth rate diversity interactions: F ≤ 1.28, P ≥ 0.27 and F ≤ 1.05, P ≥ 0.35, respectively). Comparing results for abundance vs. density distinguishes between effects of each source of diversity occurring through differences in resource abundance (e.g. there being greater plant biomass in polyculture than monoculture) vs. those occurring due to changes in plant-based heterogeneity or quality. Changes in plant quality can take place due to some unknown combination of effects of plant traits (e.g. defenses or nutrients), mutualist services, the strength of competition and predation, plant mediation of the abiotic environment or habitat complexity, and potentially other factors as well. By inference then, differences in findings between density and abundance models provide insight into the mechanisms at work. If there are significant diversity effects on abundance but not density, this suggests such effects are mediated by differences in resource abundance alone. Conversely, if diversity effects on abundance are equal to those on density, this suggests that diversity effects are mediated by plant quality rather than resource abundance.

All models included the effect of plot (treated as random) to control for spatial heterogeneity. To test for diversity effects, we performed pre-planned contrasts comparing the difference in the mean of both monoculture types (of male and female, slow and fast) relative to the mean of polycultures, separately for each source of diversity. For simplicity, we only report results from these contrasts because the test of main effects in the models was not of interest because it treats monoculture types as separate levels. For this same reason, we did not test for the growth-by-sex interaction in any case. Residuals for plant biomass variables and mycorrhizal percent root colonization were normally distributed, whereas other variables required transformations; *Uroleucon*, ant, and parasitoid abundance and density were log-transformed, *Aphis* abundance and density were log- and square-root transformed, respectively and mycorrhizae abundance was arcsine-square root transformed. We report least-square means and standard errors (S.E.) from untransformed data as descriptive statistics. In all cases, results are based upon Type III sums-of-squares and all analyses were carried out in PROC MIXED, SAS ver. 9.2 [42].

Whenever a diversity effect was significant in the above models, we determined if such effects were additive or non-additive. The former are due to sampling effects, where the occurrence of genotypes with higher growth or quality is more likely at high diversity. In contrast, non-additive effects are due to interactions among genotypes leading to emergent patch-level properties that cannot be explained by genotype-specific effects [43]. Following Johnson et al. (2006) [44], we calculated plant genotype means for each consumer group at low diversity (i.e. expected values) and compared these values to the mean of each genotype at high diversity (i.e. observed values) separately for each source of diversity (across levels of the other source of diversity). To do so, we used one-way general linear models with the PROC MIXED in SAS, including plot as a random effect and genotype nested within plot. A significant difference between observed and expected values is necessarily due to non-additivity as the comparison is performed by specifying the monoculture values of each genotype, i.e. sampling effects are accounted for by including genotype-specific expected values.

### Assessing inflation of Type I Error

Because we separately test for sex diversity and growth rate diversity on three components of plant biomass (roots, shoots, total) and the density and abundance of five plant associates (two aphids, ants, parasitoids, mycorrhizae), overall we conduct 26 analyses and thus increase the chance for Type I error (“false positives” or incorrectly rejecting the null hypothesis). Rather than reduce the number of analyses and the inference from our study, we instead took measures to assess the likelihood of inflated Type I error as described by Garcia (2004) [45]. First, we performed a binomial expansion test to determine the probability of obtaining the observed number of significant results by chance alone. Second, we perform a p-plot analysis based upon the distribution of p values produced from all analyses. With this approach, the number of true null hypotheses (i.e. non-significant results) is estimated by plotting 1-p values, sorted in ascending order, versus their rank. The points corresponding to true null hypothesis (large p-values) tend to fall along a straight line passing through the origin, whose estimated slope gives an estimate of the total number of true null hypotheses, calculated as (1/slope)-1. This estimated number of true null hypotheses can then be compared against the observed number to assess inflated Type I error.

## Results

### Effects of plant sex and growth rate diversity on plant biomass

#### Total biomass

**Fig 1:**
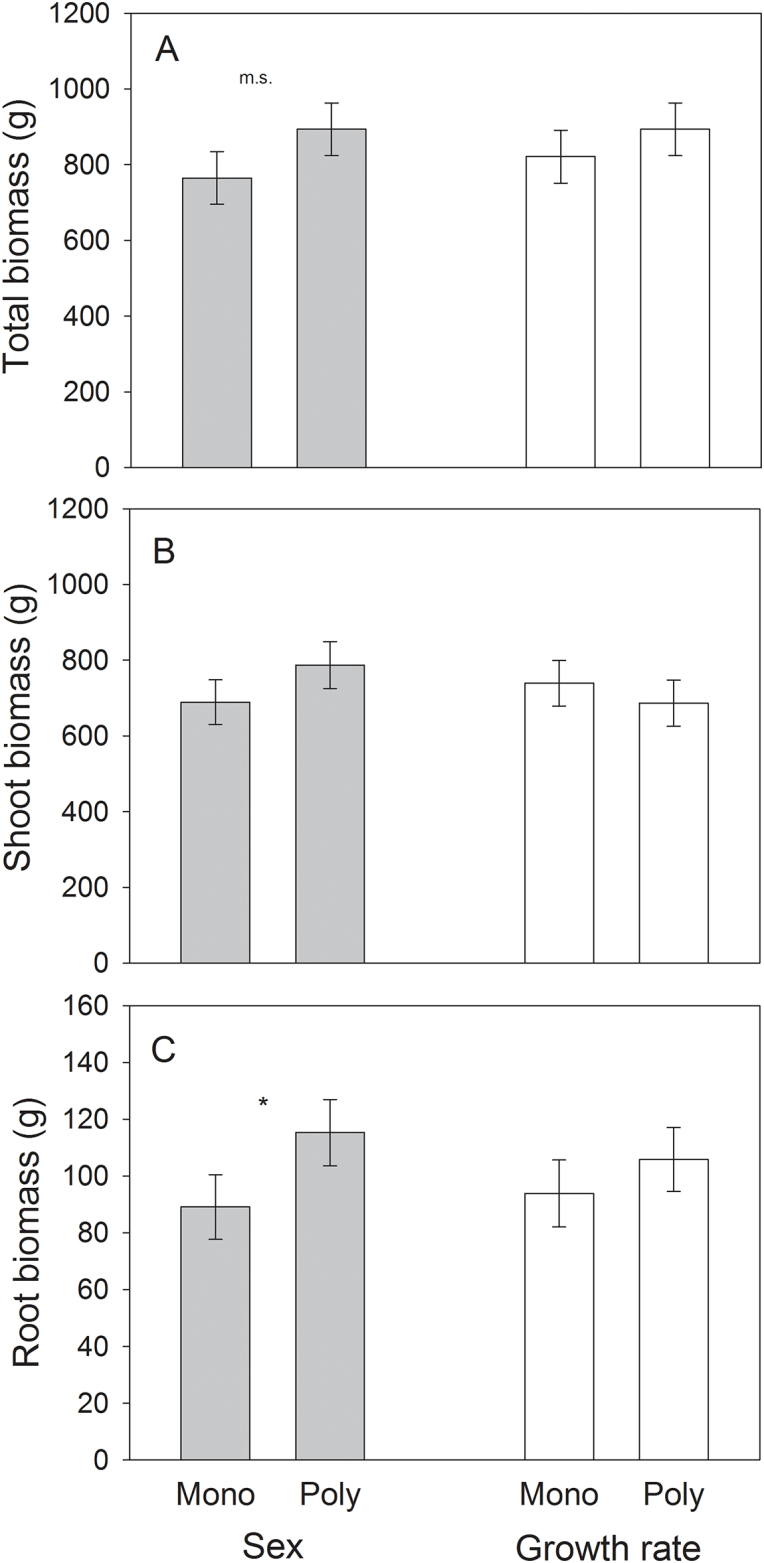
Least-square means (± S.E.) for total biomass (A), shoot biomass (B), and root biomass (C; g, dry weight in all cases) for growth rate monocultures (“mono”) and polycultures (“poly”) of fast- and slow-growing genotypes, and plant sex monocultures and polycultures of male and female genotypes of the shrub *Baccharis salicifolia*. Least-square means account for the effect of plot to control for effects of spatial heterogeneity. * = significant (*P* < 0.05); ms = marginally significant (0.05 < *P* < 0.10). The mean of sex and growth rate monocultures is the mean of male and female monocultures or of slow- and fast-growing monocultures, respectively.

There was a marginally significant effect of sex diversity on total plant biomass (Table 1), with polycultures having 20% greater total biomass (893.31 ± 69.64 g) than the mean of male and female monocultures (764.56 ± 74.06 g) (Fig 1A). In contrast, there was no effect of growth rate diversity on total biomass (Table 1; Fig 1A).

**Table 1:** Effects of plant diversity on plant biomass.

Source	Total	Shoots	Roots
	[1,56]	[1,69]	[1,56]
Sex diversity	*3*.*15(0*.*081)*	2.72(0.103)	**4.21(0.045)**
Growth diversity	0.30(0.583)	0.75(0.388)	1.07(0.306)

Results of pre-planned contrasts from general linear mixed models testing for effects of *Baccharis salicifolia* sex diversity and growth rate diversity on total plant biomass, shoot biomass, and root biomass. F-values and *P*-values (in parenthesis) correspond to contrasts testing for the difference in the mean of both monocultures relative to the mean of polycultures, separately for each source of diversity. Significant (*P* < 0.05) and marginally significant (0.05 < *P* < 0.10) results are in bold and italics, respectively. Degrees of freedom are shown in brackets below each response variable, and were the same for both tests of diversity in each case.

#### Shoot biomass

There were no effects of either sex diversity or growth rate diversity on aboveground plant biomass (Table 1), although in the former case sex polycultures (787.13 ± 61.90 g) had 14% more shoot biomass than sex monocultures (689.01 ± 59.00 g) (Fig 1B), consistent with effects on total biomass.

#### Root biomass

Sex diversity had a significant effect on root biomass (Table 1), with sex polycultures having 30% more root biomass (115.31 ± 11.67 g) relative to the mean of male and female monocultures (89.10 ± 11.40 g) (Fig 1C). Further analyses indicated that this effect tended to be non-additive (F_1,54_ = 3.50, P = 0.06), meaning that the increase in biomass in sex polycultures tended to be greater than expected based upon sampling effects alone. In contrast, there was no effect of growth rate diversity on root biomass (Table 1; Fig 1C).

### Effect of plant sex and growth diversity on associate abundance

#### Aphids

**Fig 2:**
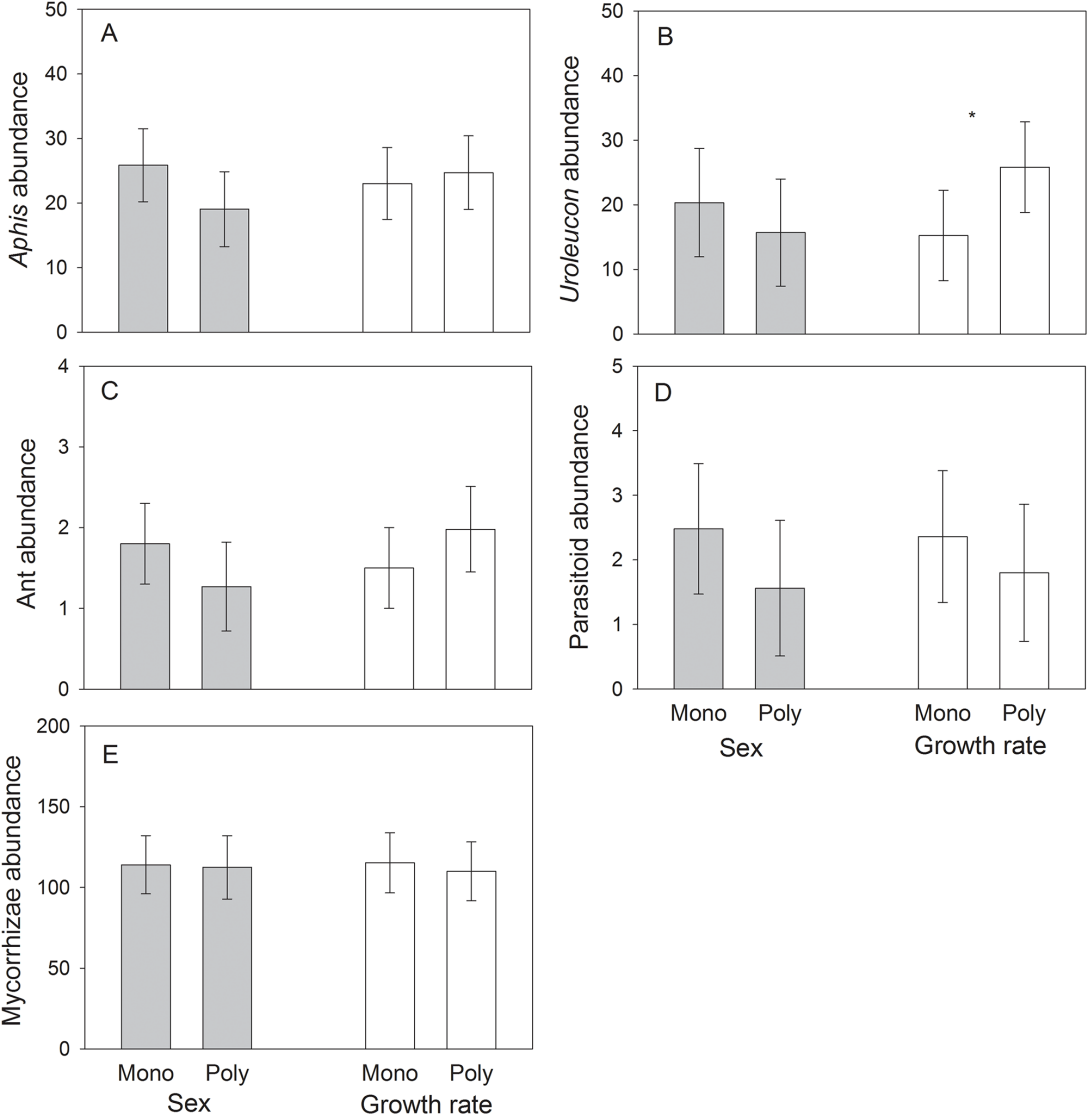
Least-square means (± S.E.) of abundance of the generalist aphid *Aphis gossypii* (A), the specialist aphid *Uroleucon macolai* (B), ants (*Linepithema humile*; C), *A*. *gossypii* parasitoids (Braconidae; D), and mycorrhizae (percent root colonization × dry root biomass; E) for growth rate monocultures (“mono”) and polycultures (“poly”) of fast- and slow-growing plant genotypes, and plant sex monocultures and polycultures of male and female genotypes of the shrub *Baccharis salicifolia*. Least-square means account for the effect of plot. * = Significant (*P* < 0.05). The mean of sex and growth rate monocultures is the mean of male and female monocultures or the mean of slow- and fast-growing monocultures, respectively.

We found no effect of sex diversity or growth rate diversity on the abundance of *Aphis* (Table 2A; Fig 2A). In contrast, there was a significant effect of growth rate diversity on *Uroleucon* abundance (Table 2A), with growth rate polycultures (25.83 ± 7.20 aphids) exhibiting a 68% greater abundance relative to the mean of slow and fast-growing monocultures (15.23 ± 7.10 aphids) (Fig 2B). Follow-up analyses indicated that this effect was additive (test of non-aditivity: F_1,64_ = 0.87, P = 0.35). In addition, the effect of sex diversity on *Uroleucon* abundance was not significant (Table 2A; Fig 2B).

**Table 2:** Effects of plant diversity on insect abundance and density.

Source	*Aphis*	*Uroleucon*	*Aphis* pars	Ants	Mycorrhizae
**A) Abundance**	[1,76]	[1,76]	[1,74]	[1,76]	[1,58]
Sex diversity	0.56(0.457)	1.02(0.314)	0.22(0.639)	1.35(0.249)	0.01(0.959)
Growth diversity	0.01(0.965)	**4.63(0.034)**	0.49(0.484)	1.83(0.178)	0.05(0.830)
**B) Density**	[1,69]	[1,69]	[1,67]	[1,69]	[1,62]
Sex diversity	**5.02(0.028)**	1.19(0.278)	0.54(0.467)	**4.87(0.030)**	0.15(0.704)
Growth diversity	0.01(0.919)	**5.46(0.022)**	0.75(0.388)	1.56(0.216)	0.01(0.993)

Results from general linear mixed models testing for effects of *Baccharis salicifolia* genotype sex and growth rate diversity on the abundance (A) and density (B) of *Aphis gossypii*, *Uruleucon macolai*, parasitoids (Braconidae) of *A*. *gossypii* (“*Aphis* pars”), ants (*Linepithema humile*), and mycorrhizae. Insect density = number of consumers per kg of shoot dry biomass; mycorrhizae abundance = proportion colonization by root dry mass; mycorrhizae density = percent root colonization (see Methods). F-values and *P*-values (in parenthesis) correspond to contrasts testing for the difference in the mean of both monocultures relative to the mean of polycultures, separately for each source of diversity. Significant (*P* < 0.05) results are in bold. Degrees of freedom are shown in brackets below each response variable, and were the same for both tests of diversity in every case.

#### Ants and parasitoids

There were no significant effects of either sex diversity or growth rate diversity on ant abundance (Table 2A; Fig 2C) or on the abundance of parasitic wasps attacking *Aphis* (Table 2A; Fig 2D).

#### Mycorrhizae

We found no effect of either sex diversity or growth rate diversity on the abundance of mycorrhizae (Table 2A; Fig 2E).

### Effect of plant sex and growth diversity on associate density

#### Aphids

**Fig 3:**
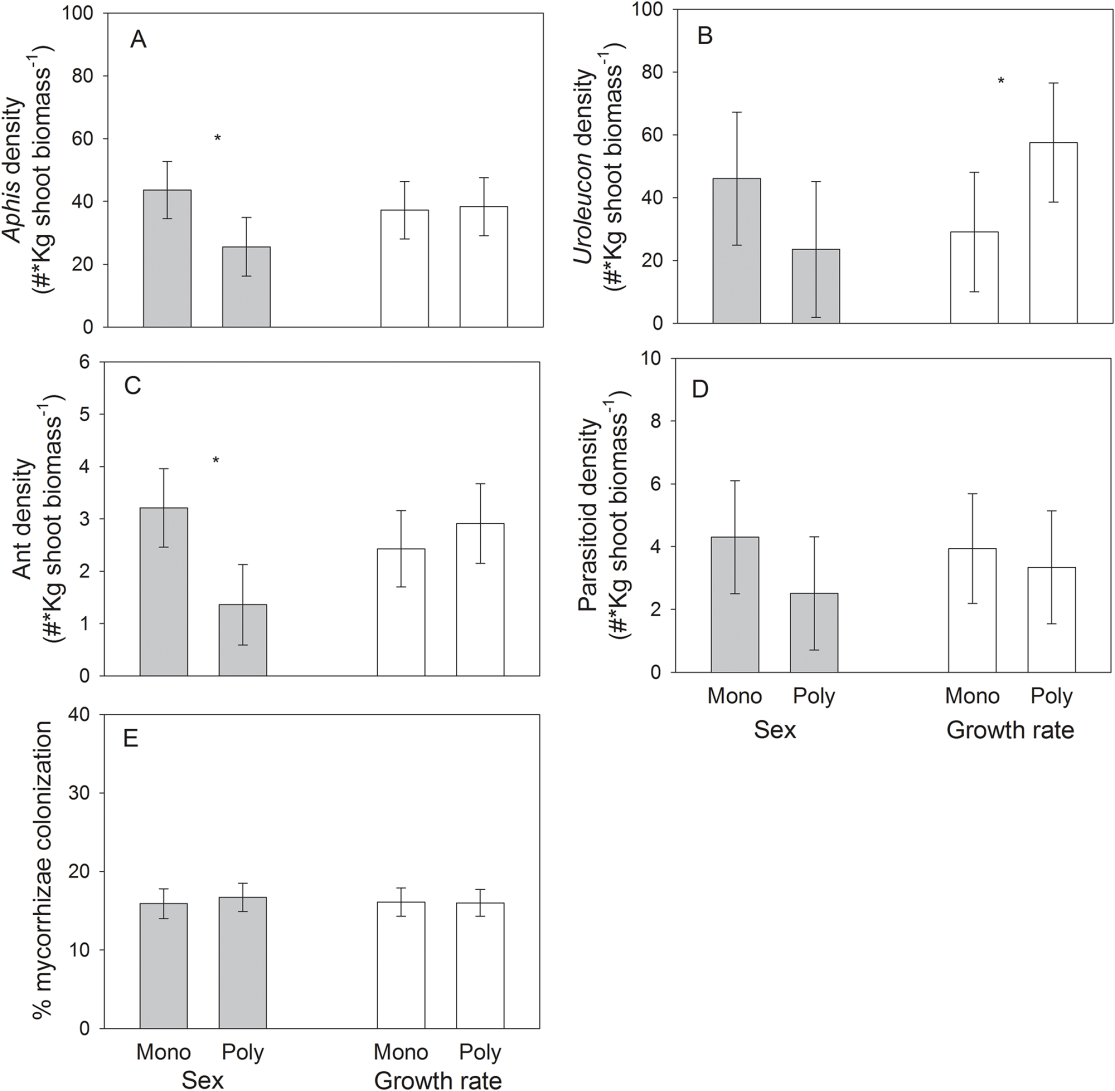
Least-square means (± S.E.) of density (# insects per kg of dry shoot biomass) of the generalist aphid *Aphis gossypii* (A), the specialist aphid *Uroleucon macolai* (B), ants (*Linepithema humile*; C), *A*. *gossypii* parasitoids (Braconidae; D), and mycorrhizal percent root colonization (E) for growth rate monocultures (“mono”) and polycultures (“poly”) of fast- and slow-growing plant genotypes, and plant sex monocultures and polycultures of male and female genotypes of the shrub *Baccharis salicifolia* Least-square means account for the effects of plot. * = Significant (*P* < 0.05). The mean of sex and growth rate monocultures is the mean of male and female monocultures or the mean of slow- and fast-growing monocultures, respectively.

We found a significant effect of plant sex diversity on *Aphis* density (Table 2B; Fig 3A), with sex polycultures (25.53 ± 9.31 aphids/kg) exhibiting a 40% lower density of this aphid than sex monocultures (43.62 ± 9.11 aphids/kg) (Fig 3A). This negative effect, however, was additive (F_1,66_ = 1.98, P = 0.16), i.e. driven by sampling effects. There was no effect of growth rate diversity on *Aphis* density (Table 2B; Fig 3A). On the other hand, we found instead a significant effect of growth rate diversity but no effect of sex diversity on *Uroleucon* density (Table 2B), where growth rate polycultures (57.53 ± 19.52 aphids/kg) exhibited a 98% greater density than monocultures (29.05 ± 19.05 aphids/kg) (Fig 3B). Such effect, however, was additive (F_1,61_ = 0.33, P = 0.56).

#### Ants and parasitoids

We found a significant effect of plant sex diversity on the density of ants (Table 2B), with sex polycultures (1.36 ± 0.77 ants/kg) exhibiting on average a 58% lower density of ants than sex monocultures (3.21 ± 0.75 ants/kg) (Fig 3C). This negative effect of diversity was non-additive (F_1,62_ = 4.65, P = 0.03). In contrast, there was no effect of growth rate diversity on ant density (Table 2B; Fig 3C). In addition, there were no effects of either sex diversity or growth diversity on the abundance of parasitic wasps attacking *Aphis* (Table 2B; Fig 3D).

#### Mycorrhizae

We found no effect of either plant sex diversity or growth rate diversity on mycorrhizal percent root colonization (Table 2B; Fig 3E).

### Assessing inflation of Type I Error

The probability of obtaining 5 significant results from 26 analyses is extremely low based upon the binomial expansion test (*p* = 0.007). Based upon a p-plot analysis (S2 Table; S2 Fig), the estimated number of true null hypotheses (non-significant results) is 21 when the regression slope is fit to only the non-significant (*p* > 0.05) p values, which qualitatively appear to fall along a straight line passing through the origin, whereas the significant (*p* < 0.05) p values qualitatively appear to follow a different slopes. However, the estimated number of true null hypotheses is 22 if the regression line is fit to all p values (the most conservative approach). These estimates in turn correspond to the 21 non-significant results observed in our study, suggesting a minimal increase in Type I error.

## Discussion

We provide novel evidence for the influence of intra-specific diversity in two axes of plant phenotypic variation, sex and growth rate, on plant growth and associated consumers. Specifically, we compared monocultures of male or female genotypes to sexual mixtures, and monocultures of fast- or slow-growing genotypes to growth-rate mixtures. Sexual diversity increased plant growth and reduced the density of the dietary generalist aphid *Aphis* and its associated aphid-tending ants, but had no effect on this aphid’s parasitoids, the specialist aphid *Uroleucon* or mycorrhizae. In contrast, growth rate diversity did not influence plant growth or any plant associates except for the specialist aphid *Uroleucon*, which exhibited higher abundance and density in growth rate mixtures. These findings indicate that plant diversity effects on consumers are contingent upon the source of plant trait variation and that such effects may vary both within and among higher trophic levels.

We found that sex diversity but not growth rate diversity influenced plant growth. In particular, the positive effect of sex diversity on root biomass and shoot biomass was non-additive (the later marginally significant), suggesting incomplete niche overlap (and thus weaker competition) or facilitation between sexes (but not among genotypes of varying growth rate). Because *B*. *salicifolia* genotypes of differing sexes did not vary in growth, we conclude that sex diversity effects on biomass were not mediated by growth-related traits. Similarly, both sex (male vs. female) and growth (fast vs. slow) had effects of similar magnitude on mycorrhizal colonization (S1 Table), suggesting that this also was not the factor underlying the effects of sex diversity on plant growth. Regardless of the specific mechanism at work, we emphasize the importance of identifying key traits or axes of variation in correlated traits to gain a predictive understanding of plant intra-specific diversity effects on plant performance. For example, differences in resource acquisition (e.g. timing and uptake levels) among genotypes with contrasting growth rates (e.g. [22]) could help explain diversity or associational effects on plant-plant interactions.

Plant diversity effects are presumably mediated by variation in plant traits influencing plant-plant interactions (and thus growth) and associate faunas. Previous analyses from this experiment indicated that the selected fast- and slow-growing *B*. *salicifolia* genotypes varied substantially in growth, and such effects in turn influenced aphids, ants, and parasitoids (S1 Table). In addition, while male and female genotypes did not vary in growth, we found sex-based differences in consumer density indicating that plant sexes vary in quality to herbivores and higher trophic levels (S1 Table). It then follows that these genetic effects should underlie concomitant effects of sex and growth diversity. Accordingly, we found differences between male and female genotypes in *Aphis* and ant density (S1 Table) and in turn effects of sex diversity on these consumers. However, in other cases we did not find a correspondence between genotypic effects and diversity effects. For example, fast- and slow-growing genotypes differed in growth rate but this did not translate into growth diversity effects on plant biomass, while male and female genotypes did not differ in growth rate, but there were sex diversity effects on plant biomass. Similar inconsistencies were observed for genotype identity vs. diversity effects on *Uroleucon* and mycorrhizae. Taken together, these findings indicate that genotype identity effects are not necessarily predictive of diversity effects, presumably due to genotype and trait interactions which complicate translating identity effects directly to diversity effects [18].

Our findings emphasize the context-dependency of plant diversity effects on higher trophic levels as a function of the source of plant trait variation [11,46] and consumer traits [47,48]. For example, we found contrasting effects of each source of diversity on the two aphids. Sex (but not growth rate) diversity negatively influenced the generalist aphid *Aphis*, with cascading negative effects on ants (but not parasitoids). In contrast growth rate (but not sex) diversity positively affected the specialist aphid *Uroleucon*. Accordingly, different axes of trait variation cascaded up to higher trophic levels in unique ways.

The fact that sex diversity increased plant growth but reduced *Aphis* density is surprising based upon past studies and plant defense theory. The negative effect of sex diversity on *Aphis* density suggests lower plant quality (e.g. due to higher plant defenses, greater predation, etc.) in sex mixtures, even though such mixtures demonstrated faster growth (relative to monocultures). This result challenges previous work showing higher plant quality (Resource Availability Hypothesis) [13,23] and increased herbivory (Plant Vigor Hypothesis) [49] for fast-growing plants. Alternatively, sex diversity may have reduced *Aphis* performance indirectly through direct negative effects on mutualist ants. Along these lines, Moreira and Mooney (2013) investigated the interactive effects of *B*. *salicifolia* genotypic diversity (one vs. four randomly selected genotypes) and ants (control vs. exclusion) on *Aphis*. This study found genotypic diversity had no effect on plant growth, increased *Aphis* abundance, but did not mediate (interact with) the top-down effects of aphid-tending ants. While these results for genotypic diversity suggest that sexual diversity may also not alter ant protection of aphids, a definitive answer would require an experiment measuring the effects of sex diversity on *Aphis* both in the presence and absence of ants.

In contrast to *Aphis*, we found a positive effect of growth diversity on both the abundance and density of the specialist aphid *Uroleucon*. In particular, a positive effect on density indicates that growth rate polycultures boosted the abundance of this aphid through changes in habitat heterogeneity or increased plant quality. We note that the magnitude of the effect of growth diversity on *Uroleucon* density was greater than for abundance despite the fact that a stronger effect would be expected on abundance since this variable responds to both resource abundance and quality. These findings, combined with the fact that growth diversity did not influence plant biomass, indicates that effects of growth diversity on *Uroleucon* through changes in resource abundance were lacking or weak at the most.

### Conclusions

Our results indicate that different sources of plant genetic diversity operate via different mechanisms and may exert contrasting effects on plant growth and consumers. Therefore, we emphasize the importance and need of manipulating known axes of plant genetic variation or genetically-based traits in order to gain a predictive understanding of plant intra-specific diversity effects [15,18]. Future work should involve the use of plant genetic lines that vary in target traits and use experimental designs that allow both the independent and combined effects of such traits to be examined. In doing so, we will move from a descriptive realm confined to addressing the presence or magnitude of plant genetic diversity effects towards identifying and assessing the relative importance of independent traits or axes of correlated traits.

## Supporting information

S1 Fig Experimental design. Experimental design for the treatment combinations of growth diversity crossed with sex diversity. Each treatment combination consisted of a pair of plants and was replicated once in each of 10 plots, with the planting location within plot being randomized such that each plot represented a randomized complete block. Plants were drawn from a pool of 14 genotypes consisting of 7 males and 7 females and 8 slow-growing and 6 fast-growing genotypes. Each of the 10 replicates of each treatment combination consisted of a unique genotypic pair.
(TIF)

S2 Fig P-plot for assessment of Type I error (after Garcia 2004). The number of true null hypotheses (i.e. non-significant results) is estimated by plotting 1-p values, sorted in ascending order, versus their rank (listed in S2 Table). The points corresponding to true null hypothesis (large p-values) tend to fall along a straight line passing through the origin, whose estimated slope gives an estimate of the number of true null hypotheses, calculated as (1/slope)-1. Significant (p < 0.05) p values are shown with hollow circles, non-significant (p > 0.05) p values are shown with filled circles. The best-fit line passing through the origin and the non-significant p values (slope = 0.0457, solid line) provides an estimate of 21 true null hypotheses (non-significant results). The best-fit line passing through the origin and all p values (slope = 0.0432, dashed line) provides an estimate of 22 true null hypotheses. The observed number of true null hypotheses was 21 out of 26 total tests (S2 Table).
(TIF)

S1 Table Plant genotype variation in plant biomass and consumers. Differences in plant biomass (total, above- and below-ground, g), consumer abundance, and consumer density (# of insects or hyphae / kg of shoot biomass) between male and female, as well as between slow- and fast-growing genotypes of *Baccharis salicifolia* reported in Abdala-Roberts et al. (2016). Consumers included the generalist aphid *Aphis gossypii* (“*Aphis*”), specialist aphid *Uroleucon macolai* (“*Uroleucon*”), parasitoids (Braconidae) attacking *A*. *gossypii* (“parasitoids”), argentine ants (*Linepithema humile*, “ants”), and mycorrhizae. Values are least-square means (± S.E.) from general linear mixed models testing for effects of sex, growth rate, their interaction, and plot (random), using data from both monocultures and polycultures (Abdala-Roberts et al. in review). Based on results from these models, we specify whether differences between levels of each factor were significant (*P < 0.05, **P < 0.01, ***P < 0.001), marginally significant (“ms”: 0.05 < P < 0.10) or not significant (“ns”: P ≥ 0.10).
(DOCX)

S2 Table Compiled values for construction of P-plots. Compiled values for p, p-1 and ranks for construction of p-plot and assessment of inflated Type I error (after Garcia 2004).
(DOCX)
