# Supplementary figures and images for: Traits underlying community consequences of plant intra-specific diversity
Source: PLoS One. 2017 Sep 8;12(9):e0183493. doi: 10.1371/journal.pone.0183493 (PMC5590834; doi:10.1371/journal.pone.0183493)

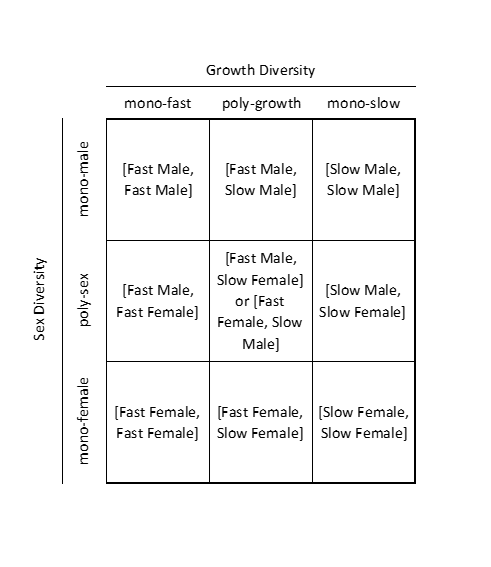

Supplement: S1 Fig — Experimental design for the treatment combinations of growth diversity crossed with sex diversity. Each treatment combination consisted of a pair of plants and was replicated once in each of 10 plots, with the planting location within plot being randomized such that each plot represented a randomized complete block. Plants were drawn from a pool of 14 genotypes consisting of 7 males and 7 females and 8 slow-growing and 6 fast-growing genotypes. Each of the 10 replicates of each treatment combination consisted of a unique genotypic pair. (TIF) [file pone.0183493.s001.tif]

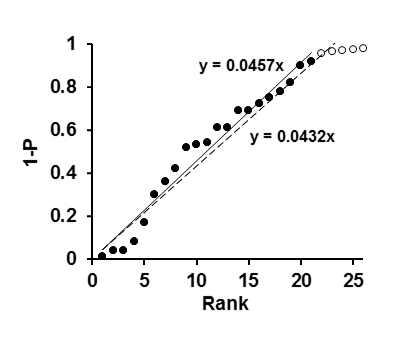

Supplement: S2 Fig — The number of true null hypotheses (i.e. non-significant results) is estimated by plotting 1-p values, sorted in ascending order, versus their rank (listed in S2 Table). The points corresponding to true null hypothesis (large p-values) tend to fall along a straight line passing through the origin, whose estimated slope gives an estimate of the number of true null hypotheses, calculated as (1/slope)-1. Significant (p < 0.05) p values are shown with hollow circles, non-significant (p > 0.05) p values are shown with filled circles. The best-fit line passing through the origin and the non-significant p values (slope = 0.0457, solid line) provides an estimate of 21 true null hypotheses (non-significant results). The best-fit line passing through the origin and all p values (slope = 0.0432, dashed line) provides an estimate of 22 true null hypotheses. The observed number of true null hypotheses was 21 out of 26 total tests (S2 Table). (TIF) [file pone.0183493.s002.tif]
