# Supplementary material for: Traits underlying community consequences of plant intra-specific diversity
Source: PLoS One. 2017 Sep 8;12(9):e0183493. doi: 10.1371/journal.pone.0183493 (PMC5590834; doi:10.1371/journal.pone.0183493)
Supplement: S1 Table — Differences in plant biomass (total, above- and below-ground, g), consumer abundance, and consumer density (# of insects or hyphae / kg of shoot biomass) between male and female, as well as between slow- and fast-growing genotypes of Baccharis salicifolia reported in Abdala-Roberts et al. (2016). Consumers included the generalist aphid Aphis gossypii (“Aphis”), specialist aphid Uroleucon macolai (“Uroleucon”), parasitoids (Braconidae) attacking A. gossypii (“parasitoids”), argentine ants (Linepithema humile, “ants”), and mycorrhizae. Values are least-square means (± S.E.) from general linear mixed models testing for effects of sex, growth rate, their interaction, and plot (random), using data from both monocultures and polycultures (Abdala-Roberts et al. in review). Based on results from these models, we specify whether differences between levels of each factor were significant (*P < 0.05, **P < 0.01, ***P < 0.001), marginally significant (“ms”: 0.05 < P < 0.10) or not significant (“ns”: P ≥ 0.10). (DOCX) [file pone.0183493.s003.docx]

**Table S1.** Differences in plant biomass (total, above- and below-ground, g), consumer abundance, and consumer density (# of insects or hyphae / kg of shoot biomass) between male and female, as well as between slow- and fast-growing genotypes of *Baccharis salicifolia* reported in Abdala-Roberts et al. (2016). Consumers included the generalist aphid *Aphis gossypii* (“*Aphis*”), specialist aphid *Uroleucon macolai* (“*Uroleucon*”), parasitoids (Braconidae) attacking *A. gossypii* (“parasitoids”), argentine ants (*Linepithema humile*, “ants”), and mycorrhizae. Values are least-square means (± S.E.) from general linear mixed models testing for effects of sex, growth rate, their interaction, and plot (random), using data from both monocultures and polycultures (Abdala-Roberts et al. in review). Based on results from these models, we specify whether differences between levels of each factor were significant (*P < 0.05, **P < 0.01, ***P < 0.001), marginally significant (“ms”: 0.05 < P < 0.10) or not significant (“ns”: P ≥ 0.10).

| Variable | **Sex** | | |  | **Growth rate** | | |
| --- | --- | --- | --- | --- | --- | --- | --- |
|  | **Male** | **Female** | **Signific.** |  | **Fast** | **Slow** | **Signific.** |
| Total biomass | 411.81 ± 31.98 | 417.46 ± 31.80 | ns |  | 541.19 ± 32.26 | 288.08 ± 31.54 | *** |
| Shoot biomass | 354.56 ± 26.80 | 366.05 ± 26.88 | ns |  | 469.15 ± 26.89 | 251.45 ± 26.79 | *** |
| Root biomass | 48.62 ± 4.69 | 50.98 ± 4.58 | ns |  | 65.10 ± 4.72 | 34.55 ± 4.57 | *** |
| *Aphis* abundance | 30.26 ± 5.08 | 17.78 ± 5.09 | ** |  | 29.24 ± 5.09 | 18.80 ± 5.08 | ms |
| *Aphis* density | 112.94 ± 21.37 | 59.33 ± 21.41 | ** |  | 76.13 ± 21.36 | 96.09 ± 21.35 | ns |
| *Uroleucon* abundance | 16.38 ± 8.02 | 21.39 ± 8.01 | ms |  | 22.49 ± 8.39 | 16.78 ± 8.37 | ns |
| *Uroleucon* density | 109.56 ± 49.59 | 83.66 ± 49.56 | ns |  | 78.33 ± 49.58 | 114.89 ± 48.50 | ns |
| Parasitoid abundance | 2.85 ± 0.90 | 1.46 ± 0.95 | * |  | 2.39 ± 0.95 | 1.91 ± 0.95 | ns |
| Parasitoid density | 10.85 ± 4.05 | 5.03 ± 4.06 | * |  | 8.95 ± 3.05 | 6.89 ± 3.05 | ns |
| Ant abundance | 2.16 ± 0.46 | 1.07 ± 0.46 | ** |  | 2.17 ± 0.46 | 1.06 ± 0.46 | *** |
| Ant density | 8.45 ± 1.32 | 2.79 ± 1.33 | *** |  | 6.11 ± 1.21 | 5.25 ± 1.20 | ns |
| Mycorrhizae abundance | 45.65 ± 6.97 | 62.95 ± 7.17 | ms |  | 63.66 ± 6.84 | 44.95 ± 7.25 | * |
| Mycorrhizae density | 0.14 ± 0.013 | 0.18 ± 0.015 | ms |  | 0.13 ± 0.013 | 0.18 ± 0.014 | * |
