# Supplementary material for: Traits underlying community consequences of plant intra-specific diversity
Source: PLoS One. 2017 Sep 8;12(9):e0183493. doi: 10.1371/journal.pone.0183493 (PMC5590834; doi:10.1371/journal.pone.0183493)
Supplement: S2 Table — Compiled values for p, p-1 and ranks for construction of p-plot and assessment of inflated Type I error (after Garcia 2004). (DOCX) [file pone.0183493.s004.docx]

**Table S2.**  Compiled values for p, p-1 and ranks for construction of p-plot and assessment of inflated Type I error (after Garcia 2004).

| p | 1-p | rank |
| --- | --- | --- |
| 0.022 | 0.978 | 26 |
| 0.028 | 0.972 | 25 |
| 0.03 | 0.97 | 24 |
| 0.034 | 0.966 | 23 |
| 0.045 | 0.955 | 22 |
| 0.081 | 0.919 | 21 |
| 0.103 | 0.897 | 20 |
| 0.18 | 0.82 | 19 |
| 0.22 | 0.78 | 18 |
| 0.25 | 0.75 | 17 |
| 0.28 | 0.72 | 16 |
| 0.31 | 0.69 | 15 |
| 0.31 | 0.69 | 14 |
| 0.388 | 0.612 | 13 |
| 0.39 | 0.61 | 12 |
| 0.46 | 0.54 | 11 |
| 0.47 | 0.53 | 10 |
| 0.48 | 0.52 | 9 |
| 0.58 | 0.42 | 8 |
| 0.64 | 0.36 | 7 |
| 0.7 | 0.3 | 6 |
| 0.83 | 0.17 | 5 |
| 0.919 | 0.081 | 4 |
| 0.96 | 0.04 | 3 |
| 0.96 | 0.04 | 2 |
| 0.99 | 0.01 | 1 |
